# Do men regret prostate biopsy: Results from the PiCTure study

**DOI:** 10.1186/s12894-016-0194-y

**Published:** 2017-01-26

**Authors:** Catherine Coyle, Eileen Morgan, Frances J. Drummond, Linda Sharp, Anna Gavin

**Affiliations:** 1 Public Health Agency, Belfast, Northern Ireland; 2 Northern Ireland Cancer Registry, Queen’s University, Belfast, Northern Ireland; 3 National Cancer Registry Ireland, Cork, Ireland; 4 Department of Epidemiology and Public Health, University College Cork, Cork, Ireland; 5 Institute of Health & Society, Newcastle University, Newcastle, UK

**Keywords:** Prostate, Biopsy, Regret

## Abstract

**Background:**

Understanding men’s experience of prostate biopsy is important as the procedure is common, invasive and carries potential risks. The psychological aspects of prostate biopsy have been somewhat neglected. The aim of this study was to explore the level of regret experienced by men after prostate biopsy and identify any associated factors.

**Methods:**

Men attending four clinics in Republic of Ireland and two in Northern Ireland were given a questionnaire to explore their experience of prostate biopsy. Regret was measured on a Likert scale asking men how much they agreed with the statement “It [the biopsy] is something I regret.”

**Results:**

Three hundred thirty-five men responded to the survey. The mean age was 63 years (SD ±7 years). Three quarters of respondents (76%) were married or co-habiting, and (75%) finished education at primary or secondary school level. For just over two thirds of men (70%) their recent biopsy represented their first ever prostate biopsy. Approximately one third of men reported a diagnosis of cancer, one third a negative biopsy result, and the remaining third did not know their result. Two thirds of men reported intermediate or high health anxiety. 5.1% of men agreed or strongly agreed that they regretted the biopsy.

**Conclusions:**

Level of regret was low overall. Health anxiety was the only significant predictor of regret, with men with higher anxiety reporting higher levels of regret than men with low anxiety (OR = 3.04, 95% CI 1.58, 5.84).

Men with high health anxiety may especially benefit from careful counselling before and after prostate biopsy.

**Electronic supplementary material:**

The online version of this article (doi:10.1186/s12894-016-0194-y) contains supplementary material, which is available to authorized users.

## Background

Prostate biopsy is an invasive test that involves rectal insertion of an ultrasound probe to diagnose cancer of the prostate. It is usually prompted by a raised Prostate Specific Antigen (PSA), prostatic symptoms, an abnormal digital rectal examination (DRE) or a combination of these. The incidence of prostate cancer has until recently increased in most developed countries [1] and has the potential to increase further in future decades [2]. While acknowledging that predictions can be uncertain and that the ongoing debate about the benefits of screening for prostate cancer may also affect incidence, given population growth and the growing proportion of older people in the population, it is possible that the absolute number of biopsies will increase further. Prostate biopsy can be difficult for men to tolerate, and commonly results in physical side effects [3, 4] including bleeding, pain, urinary retention and infection. While the physical side effects have been well investigated, the psychological impact of prostate biopsy has been somewhat neglected [5, 6].

Decision-related regret is a negative emotion associated with thinking about a choice one has made or is about to make [7]. Evidence has grown which shows that men who choose different treatment options for prostate cancer report differing levels of regret, and the factors which predict regret have become a focus of investigation [8]. Previous studies of men with prostate cancer suggest that the demographic factors of: older age [8], being single [8–10] and lower educational attainment [10, 11] were associated with higher levels of treatment regret. Clinically, those experiencing treatment-related complications/side effects [8], with better pre-operative erectile function, post-operative incontinence, longer time from surgery to survey [8], and trait anxiety [9], were associated with higher levels of treatment regret. Decisional regret with respect to prostate biopsy does not appear to have been investigated.

The aim of this study was to investigate, for the first time, levels of decisional regret in men undergoing prostate biopsy and the factors associated with this. The hypothesis was that regret would be low and would not represent a significant burden for men undergoing prostate biopsy. The rationale for this was that men are likely to feel reassurance irrespective of the biopsy result; a negative biopsy result provides relief, and a positive result justifies the decision to proceed with biopsy.

## Methods

### Setting

The study took place on the island of Ireland, which comprises the Republic of Ireland (RoI) and Northern Ireland (NI). Four of the eight rapid access clinics (RAC) in the RoI public healthcare system agreed to take part in the study. In NI, two of the five Health and Social Care Trusts which are part of the public-funded NHS participated.

Ethical approval for the study was obtained for the four participating RoI hospitals and from the office for Research Ethics in Northern Ireland.

### Recruitment

Men were recruited between November 2012 and December 2013. They were eligible to participate if they were undergoing prostatic biopsy as a result of a raised PSA level and/or an abnormal DRE, were over eighteen years of age, could understand English, were usually resident in either the RoI or NI, and were deemed well enough by their medical teams to complete a questionnaire, and in particular, had no cognitive impairment. They were ineligible if they had a previous diagnosis of prostate cancer.

Two methods of recruitment were used due to differing ethical and data protection requirements in the participating hospitals. In two hospitals in the RoI, a study information leaflet was sent to all men with their prostate biopsy appointment. Between four and six weeks post-biopsy these men were sent a questionnaire pack, with two reminder letters sent to non-responders at fortnightly intervals.

In the remaining hospitals all men, when attending for their prostate biopsy result, were given a questionnaire pack by nurse specialists. These men received no written reminders. In total 811 men were given/sent a questionnaire.

### Questionnaire

The questionnaire captured socio-demographic information including date of birth, marital status, and educational attainment.

Men were asked about their urinary symptoms prior to first PSA test, and also questions about their route to PSA testing. They were also asked if, prior to biopsy, they had experienced urinary symptoms or erectile dysfunction. Information was requested regarding preparation for biopsy and number of cores taken.

Eighteen statements about men’s feelings about prostate biopsy (both positive and negative) were developed based on discussions with prostate cancer survivor groups. Men were asked to record their level of agreement, on a 4-point Likert scale ranging from “strongly agree” to “strongly disagree”, with each of the statements. Regret was measured with the statement “It [the biopsy] is something I regret.”

Information was sought on the biopsy result. Men were asked questions about their experience of specific physical side effects following their most recent biopsy (fever, bleeding, pain, erectile dysfunction and urinary retention). Questions enquired about the severity, duration and need for treatment where these occurred.

The questionnaire was pretested with 24 men attending three prostate cancer groups in the RoI, and modified accordingly.

A copy of the questionnaire is included as Additional file 1.

### Statistical analysis

As there is limited literature on regret of biopsy, the factors investigated were chosen based on the literature on regret after cancer treatment decisions, and are largely explorative. These included socio-demographic variables, health anxiety, clinical variables and physical side effects.

Ordinal logistic regression was used to explore relationships between these variables and regret. An initial univariate analysis was conducted. Physical side effects (fever, bleeding, pain, erectile dysfunction and urinary retention) were analysed as both ‘any side effect’, and also individually for an association with regret. Variables significant at *p* < 0.10 were added simultaneously to a multivariate model and likelihood ratio tests obtained to assess whether each variable should be included in the final model (*p* ≤ 0.05). The proportional odds assumption was checked using a likelihood ratio test.

Chi-squared tests were used to investigate differences in characteristics between those who responded to the question on regret and those who did not.

Statistical analyses were conducted using STATA release 11 (StataCorp, College Station, TX).

## Results

Three hundred thirty-five men responded to the survey (response rate 41%). The demographic features of respondents are shown in Table 1. The mean age was 63 years. Three-quarters of respondents (76%) were married or co-habiting, and 75% finished education at primary or secondary school level. 38% were working and 39% retired at the time of survey completion. For just over two thirds (70%) of men, their recent biopsy represented their first ever biopsy. Approximately one third of men reported a diagnosis of cancer, one third a negative biopsy result, and the remaining third did not know their result. 88% of men reported experiencing one or more physical side effects, most commonly bleeding (reported by 80%). Just over half (53.4%) of respondents reported that these were the same or better than expected. Two thirds of men reported high or intermediate health anxiety.

**Table 1:** Socio-demographic and clinical characteristics of participants

	Frequency
	n (%)
Total	335 (100.0)
Socio-demographic variables	
*Age at questionnaire completion*	
< 65 years	192 (57.3)
≥ 65 years	140 (41.8)
Missing	3 (0.9)
*Marital status*	
Married/partnership	254 (75.8)
Other	80 (23.9)
Missing	1 (0.3)
*Highest education level completed*	
Primary/ secondary school	250 (74.6)
Third level	78 (23.3)
Missing	7 (2.1)
*Employment status*	
Working	128 (38.4)
Retired	130 (38.8)
Other	66 (19.7)
Missing	11 (3.3)
*Health anxiety*	
Low	112 (33.4)
Intermediate	148 (44.2)
High	74 (22.1)
Missing	1 (0.3)
Clinical variables	
*Given choice of first PSA*	
Yes	243 (72.5)
No	63 (18.8)
Missing	29 (8.7)
*Pre-biopsy symptoms*	
Incontinence	
None/mild	244 (72.8)
Moderate/severe	53 (15.8)
Missing	38 (11.3)
Erectile dysfunction	
None/mild	239 (71.3)
Moderate/severe	75 (22.4)
Missing	21 (6.3)
*Symptoms at time of PSA test*	
Asymptomatic	188 (56.1)
Symptomatic	119 (35.5)
Other/Missing	28 (8.4)
Enough information pre-biopsy	
Yes received enough	270 (80.6)
Yes but would have liked more	25 (7.5)
No but did not want/need any	6 (1.8)
No but would have liked some	13 (3.9)
Missing	21 (6.3)
Biopsy result	
Negative	109 (32.5)
Positive	118 (35.2)
Don’t know	108 (32.2)
Expectations of side-effects	
Same as expected	117 (34.9)
Worse than expected	46 (13.7)
Not as bad as expected	62 (18.5)
Did not have side-effects	97 (29.0)
Missing	13 (3.9)
Number of previous biopsies (including most recent biopsy)	
One	234 (69.9)
More than one	83 (24.8)
Missing	18 (5.4)
Physical Side-effects from biopsy	
*Any side-effect*Z^*a*^	
Yes	295 (88.1)
No	38 (11.3)
Missing	2 (0.6)

^a^Fever, bleeding, pain, erectile dysfunction and urinary retention

Of the 335 men, 11.9% did not respond to the question on regret and thus were excluded from further analysis. More of the men who completed the regret question were married or in a partnership (77.9% vs 62.5%, *p* = 0.03) and had third level education (26.7% vs. 2.5%, *p* = 0.01) compared to those who did not.

Of the 295 men who answered the regret question, 5.1% agreed (1.4%) or strongly agreed (3.7%) with the statement “It [the biopsy] is something I regret”. 37.6% of men disagreed, and 57.3% strongly disagreed.

The univariate analysis found significant associations between health anxiety (*p* < 0.01) and number of previous prostate biopsies (including the most recent biopsy) (*p* = 0.03) with regret (Table 2). There was no association with individual physical side effects and regret, or with ‘any physical side effect’. In multivariate analyses, the significant association between increasing health anxiety and biopsy regret remained after adjusting for number of biopsies. After adjusting for health anxiety, the number of biopsies was no longer significantly associated with regret (Table 3).

**Table 2:** Results of univariate ordinal logistic regression testing for association of predictor variables with regret post-biopsy: odds ratios (OR) with 95% confidence intervals (CI) and *p*- values

	Frequency	Univariate Analysis	
	n (%)	OR (95% CI)^a^	*p*-value*
Total	295 (100.0)		
Socio-demographic variables			
*Age at questionnaire completion*			
<65 years	174 (59.0)	1.00	
≥65 years	118 (40.0)	0.89 (0.56, 1.42)	0.64
Missing	3 (1.0)		
*Marital status*			
Married/ partnership	229 (77..6)	1.00	
Other	65 (22.0)	0.99 (0.57, 1.71)	0.96
Missing	1 (0.3)		
*Highest education level completed*			
Primary/secondary school	211 (71.5)	1.00	
Third level	77 (26.1)	0.67 (0.40, 1.14)	0.14
Missing	7 (2.4)		
*Employment status*			
Working	114 (38.6)	1.00	
Retired	117 (39.7)	0.72 (0.43, 1.22)	
Other	55 (18.6)	1.17 (0.62, 2.19)	0.27
Missing	9 (3.1)		
*Health anxiety*			
Low	100 (33.9)	1.00	
Intermediate	132 (44.8)	1.80 (1.05, 3.10)	
High	63 (21.4)	3.18 (1.68, 6.02)	0.001
Missing	0 (0.0)		
Clinical variables			
*Given choice of first PSA*			
Yes	215 (72.9)	1.00	
No	57 (19.3)	0.86 (0.48, 1.53)	0.60
Missing	23 (7.8)		
*Pre-biopsy symptoms*			
Incontinence			
None/mild	222 (75.3)	1.00	
Moderate/severe	45 (15.3)	1.12 (0.59, 2.11)	0.73
Missing	28 (9.5)		
Erectile dysfunction			
None/mild	213 (72.2)	1.00	
Moderate/ severe	65 (22.0)	1.08 (0.62, 1.89)	0.77
Missing	17 (5.8)		
*Symptoms at time of PSA test*			
Asymptomatic	166 (56.3)	1.00	
Symptomatic	106 (35.9)	1.34 (0.83, 2.17)	0.23
Other/Missing	23 (7.8)		
Enough information pre-biopsy			
Yes received enough	238 (80.7)	1.00	
Yes but would have liked more	24 (8.1)	1.84 (0.81, 4.19)	
No but did not want/need any	5 (1.7)	2.84 (0.47, 17.27)	
No but would have liked some	12 (4.1)	1.10 (0.34, 3.54)	0.36
Missing	16 (5.4)		
Biopsy result			
Negative	106 (35.9)	1.00	
Positive	97 (32.9)	1.45 (0.84, 2.51)	
Don’t know	92 (31.2)	1.34 (0.76, 2.35)	0.38
Expectations of side-effects			
Same as expected	108 (36..6)	1.00	
Worse than expected	41 (13.9)	1.97 (0.98, 3.96)	
Not as bad as expected	54 (18.3)	1.39 (0.72, 2.68)	
Did not have side-effects	83 (28.1)	1.08 (0.60, 1.93)	0.24
Missing	9 (3.1)		
Number of biopsies			
One	209 (70.9)	1.00	
More than one	72 (24.4)	0.62 (0.36, 1.07)	0.08
Missing	14 (4.8)		
Physical Side-effects from biopsy			
*Any side-effect* ^*b*^			
Yes	263 (89.2)	1.00	
No	32 (10.9)	0.88 (0.42, 1.84)	0.74
Missing	0 (0.0)		

*p-values obtained from the likelihood ratio test

^a^The OR for ordinal logistic regression shows the likelihood of having an increased level of the outcome variable (regret) within each category of the predictor variable compared to a reference category in the predictor variable

^b^Fever, bleeding, pain, erectile dysfunction and urinary retention

**Table 3:** Results of multivariate ordinal logistic regression: multivariate model including predictor variables which had *p*-value <0.10 in univariate ordinal logistic regression - odds ratios (OR) with 95% confidence intervals (CI) and *p*- values

Variable	OR (95% CI)	*p*-values*
Number of previous biopsies (including most recent biopsy)		
One	1.00	
More than one	0.68 (0.39, 1.19)	0.17
Health anxiety		
Low	1.00	
Intermediate	1.87 (1.07, 3.26)	<0.01
High	3.04 (1.58, 5.84)	

*p-values obtained from the likelihood ratio test

## Discussion

This study found that overall levels of regret were low (5%) among men following prostate biopsy. A higher level of regret following prostate cancer treatment decisions (11–12%) has been reported in a number of published studies [8, 10, 12] suggesting the possibility of differences in how men view the decisions around biopsy and treatment. Further suggestion of the difference between biopsy and treatment regret is reflected in the different predictor variables significantly associated with regret in this study compared with the existing literature on regret following prostate cancer treatment. One theory to explain this difference and supported by our results is that these men may have received better pre-procedure counselling for prostate biopsy. This is based on more than 80% of men reporting that they received enough information before the biopsy, and less than one fifth of men had side effects worse than expected. Also, at this point more than one-third of these men did not have cancer, a fact for which they may be grateful that they had the biopsy. Further research is needed to clarify if and how predictors of regret in biopsy and treatment differ with the aim of ensuring men are appropriately prepared and counselled at each point in the prostate cancer diagnosis and treatment pathway.

The evidence base for PSA testing as a screening tool for prostate cancer – the route by which many men are referred for a prostate biopsy - includes conflicting results which has not as yet made a clear case for widespread PSA testing. However its use in Ireland [13] and elsewhere is widespread. Ransohoff and McNaughton Collins argue that this widespread use is because the system acts to make PSA attractive through positive feedback mechanisms [14]. A patient will be grateful for a negative PSA result or suspicious result followed by a negative biopsy; furthermore a positive PSA result followed by a cancer diagnosis makes the patient grateful for early detection. The clinician is also affected by positive feedback resulting from PSA testing and subsequent biopsy; we have previously shown that GPs who detect an asymptomatic prostate cancer via PSA testing were 3-times more likely to PSA test other asymptomatic men [15]. Additionally, litigation will usually only follow a cancer detected too late, not the one detected too early or which would never have caused harm.

This theory may explain why the level of regret reported by respondents in this survey was generally low; a negative biopsy result provides reassurance, and a positive result provides positive feedback that the test was worth it as the cancer has been detected.

Health anxiety was the only variable significantly associated with regret. Health anxiety can be thought of as a continuum, with hypochondriasis at the extreme. It is characterised by attentional biases towards illness-related information and cognitive biases leading to the misinterpretation of information as personally threatening and catastrophic [16]. Miles et al. [16] examined health anxiety in the context of screening for colorectal cancer and found that people with high health anxiety were less reassured following screening. In this study we found that men with higher health anxiety report higher levels of regret and, following from Miles et al. [16], it may be that men with high health anxiety were less reassured following prostate biopsy, and therefore were more likely to regret the procedure.

This study does have some limitations. The questionnaire designed to collect this data was in effect a new instrument. However it was tested for face validity and comprehension with 24 men in the RoI, and available validated tools were used within the questionnaire such as the Health Anxiety Questionnaire by Lucock and Morley, 1996. The limited sample size coupled with the low level of regret may explain in part why so few variables were associated with regret. The response rate is another limitation and we do not have any information on the characteristics of responders and non-responders, so we cannot assess participation bias. Respondents who answered the question on regret were more likely to be married or in a partnership and to have third level education than those who did not. The literature cited previously on regret after treatment decisions indicates that being married or in a partnership, and higher levels of educational attainment are associated with lower levels of regret. This suggests we may have somewhat underestimated regret in the current study.

The major strength of the study is that we were able to test a wide variety of variables for an association with regret. The inclusion of men from two jurisdictions with differing health systems increases generalizability.

The study overall can be viewed as a pilot study into regret among men following prostate biopsy and associated factors. This should raise the profile of this issue among researchers and the health community which may in turn lead to further research to explore this in other populations.

## Conclusions

In conclusion, regret is low overall shortly after a prostate biopsy. Men with high health anxiety are more likely to report higher levels of regret. These men may especially benefit from careful counselling before and after biopsy. Given the potential for the number of biopsies being performed to increase and the limited evidence-base, further research on different aspects of men’s views and experiences of biopsy would be of value.

## Additional file

Additional file 1: The PiCTure Study – Prostate Investigations in Ireland. This is a pdf version of the questionnaire used in the Republic of Ireland. (PDF 1351 kb)

## Abbreviations

DRE: Digital rectal examination
NI: Northern Ireland
PSA: Prostate specific antigen
RAC: Rapid access clinics
RoI: Republic of Ireland
