# Supplementary material for: Do men regret prostate biopsy: Results from the PiCTure study
Source: BMC Urol. 2017 Jan 26;17:11. doi: 10.1186/s12894-016-0194-y (PMC5270322; doi:10.1186/s12894-016-0194-y)
Supplement: Additional file 1: — The PiCTure Study – Prostate Investigations in Ireland. This is a pdf version of the questionnaire used in the Republic of Ireland. (PDF 1351 kb) [file 12894_2016_194_MOESM1_ESM.pdf]

# PiCTure

**Prostate Investigations: *your experience***

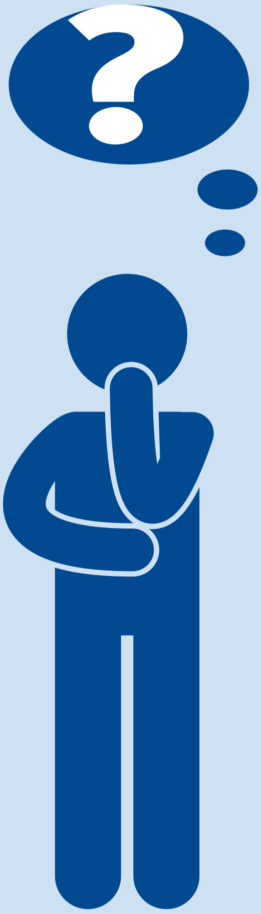

National Cancer Registry Ireland

HRB Health Research Board

PROSTATE CANCER UK

**Ref. No.**

PiCTure
Prostate Investigations: *your experience*

# *The PiCTure Study: Prostate investigations in Ireland*

***A few weeks ago you had a prostate biopsy at the hospital. We want to find out about your experience and how you have been feeling since then.***

***This questionnaire will take about 20 minutes to complete. All of the questions are important so please try to answer all the ones that apply to you. If you need help filling in the questionnaire you can ask someone you feel comfortable with to help you; please tell us if someone helped you and their relationship to you in question A1. If you would like more information about the study, please contact the project coordinator at 021-4548837. If you have any questions about your recent tests, or the results, please contact your hospital team or GP.***

***When you have filled in the questionnaire and signed the consent form, please return them both to us in the pre-paid envelope provided – no stamp is required. Thank you for your help.***

***Your questionnaire and everything you tell us will be kept strictly confidential***

# Section A About You

*Please answer each question by putting a ✓ in the box or by using the spaces provided*

**A1 a. Please fill in the date you are completing this questionnaire:**

d: ☐☐ m: ☐☐ y: ☐☐☐☐

**b. Is someone helping you to fill in this survey?** Yes: ☐ 1 No: ☐ 2

**c. *If Yes,* what is their relationship to you *(e.g. wife, son)?***

**A2 What is your date of birth?** d: ☐☐ m: ☐☐ y ☐☐☐☐

**A3 a. What is your nationality?** Irish: ☐ 1 Other: ☐ 2

**b. *If Other,* please describe:**

**A4 What is your marital status? *(Please tick ONE box)***

| | | | |
|---|---|---|---|
| Married | ☐ 1 | Separated/Divorced | ☐ 4 |
| Never married | ☐ 2 | Living with a partner/co-habiting | ☐ 5 |
| Widowed | ☐ 3 | Living in a civil partnership | ☐ 6 |

**A5 a. Do you live alone?** Yes: ☐ 1 No: ☐ 2

***If No,***

**b. how many children under 16 live with you?** ☐☐

**c. how many children 16 and older live with you?** ☐☐

**A6 a. What age were you when you finished full-time education?** ☐☐ **Years old**

**b. What is the highest level of education you have completed? *(Please tick ONE box)***

| | | | |
|---|---|---|---|
| Primary school | ☐ 1 | Third level *(e.g. college, university)* | ☐ 3 |
| Secondary school | ☐ 2 | Post graduate *(e.g. masters, doctorate)* | ☐ 4 |

**A7 a. Do you have/use any of the following? *(Please yes OR no to each)***

| | Yes | No | | Yes | No |
|---|---|---|---|---|---|
| i) A medical card | ☐ 1 | ☐ 2 | iii) Drug payment scheme | ☐ 1 | ☐ 2 |
| ii) Private health insurance | ☐ 1 | ☐ 2 | iv) GP only card | ☐ 1 | ☐ 2 |

**A8 a. Do you receive any social welfare/social security benefits or payments? *(eg. old age pension, disability allowance)***

Yes: ☐ 1 No: ☐ 2

**b. *If Yes,* please describe:**

**A9 Has your father, or any of your brothers, been diagnosed with prostate cancer?**

Yes: ☐ 1 No: ☐ 2 Don't know: ☐ 3

**A10 These questions are about how you feel about your health *(Please tick the box that best applies to you for EACH question)***

| | Not at all/Rarely | Sometimes | Often | Most of the time |
|---|---|---|---|---|
| a. If you have an ache or pain do you worry it might be caused by a serious illness? | ☐ 1 | ☐ 2 | ☐ 3 | ☐ 4 |
| b. Do you find it difficult to keep worries about your health out of your mind? | ☐ 1 | ☐ 2 | ☐ 3 | ☐ 4 |
| c. When you hear about an illness on TV or radio, does it make you think you may be suffering from that illness? | ☐ 1 | ☐ 2 | ☐ 3 | ☐ 4 |

**A11 How many people can you count on if you have serious personal problems? *(Please tick ONE box)***

None: ☐ 1 1 or 2 people: ☐ 2 3 to 5 people: ☐ 3 More than 5 people: ☐ 4

# Section B Before the Biopsy

***Before having a biopsy, most men have a PSA blood test. The first questions in this section are about your PSA tests (if any). Please answer each question by putting a ✓ in the appropriate box or using the space provided.***

**B1** **<u>Before your first biopsy,</u> did you have a PSA blood test?** Yes: ☐ 1 No: ☐ 2 Don't know: ☐ 3

**If No or Don't Know, go to B9**

**B2** **a. When was your <u>first</u> PSA blood test? *(If you don't know exactly, give your best estimate)***

d: ☐☐ m: ☐☐ y: ☐☐☐☐

**b. What was the result of your <u>first</u> PSA blood test? *(Please tick ONE box)***

Within normal range ☐ 1 Can't remember/Don't know ☐ 3

High ☐ 2 I am waiting for the result ☐ 4

**c. If you know the exact result of your <u>first</u> PSA blood test, please tell us:**

[ ] 1 Don't know ☐ 3

**d. Where did you have your <u>first</u> PSA blood test?** GP ☐ 1 Hospital ☐ 2 Other ☐ 3

**e. *If Other,* please describe** [ ]

**B3** **Thinking of your <u>first</u> PSA blood test, what best describes your experience? *(Please tick ONE box)***

I attended my GP with <u>urinary symptoms</u> *(eg. urinating frequently, blood in urine, poor flow)* and my GP did a PSA test ☐ 1

I attended my GP with <u>other symptoms</u> *(eg. back pain, joint pain)* and my GP did a PSA test ☐ 2

I had no symptoms and my <u>GP offered to test my PSA</u> as part of a general health check ☐ 3

I had no symptoms and <u>I asked my GP</u> to measure my PSA ☐ 4

I attended my GP for another reason and <u>was unaware that my PSA was being tested</u> ☐ 5

I had my first PSA test <u>at the hospital</u> ☐ 6

I had my first PSA test <u>somewhere else</u> *(eg. nurse-led clinic, screening bus or private check up)* ☐ 7

*If Somewhere Else,* please describe [ ]

**B4** **Did you feel that you were given a choice about whether you wanted to have your <u>first</u> PSA blood test?**

Yes: ☐ 1 No: ☐ 2

**B5** **Before your <u>first</u> PSA blood test, were you given information about what the test was for, what it would involve, and the pros and cons of being tested? *(Please tick ONE box)***

Yes, I was given enough information ☐ 1

Yes, but I would have liked more information ☐ 2

No, I was not given any information, but I did not want/need any ☐ 3

No, I was not given any information, but I would have liked some ☐ 4

**B6 Before your <u>first</u> PSA blood test, did the doctor or nurse explain that this test was to help find out whether you had prostate cancer?** ***(Please tick ONE box)***

Yes, the explanation was clear ☐ 1

Yes, but the explanation could have been clearer ☐ 2

No explanation was given ☐ 3

**B7 Before your <u>first</u> PSA blood test, did the doctor or nurse explain what would happen if your PSA raised or the result was abnormal?** Yes: ☐ 1 No: ☐ 2

**B8 a. Altogether, <u>how many</u> PSA blood tests have you had?** ***(If you don't know exactly, give your best estimate.)***

Number of PSA blood tests ☐ 1 Can't remember ☐ 2

**b. If you have had more than one test, how often do you have PSA blood tests?** ***(Please tick ONE box)***

Every 6 months ☐ 1 Every year ☐ 2 Every two years ☐ 3 When my doctor recommends it ☐ 4

***The following questions ask you to think about just before your first prostate biopsy***

**B9 <u>Before your first biopsy,</u> was your health?** ***(Please tick ONE box)***

Very good ☐ 1 Good ☐ 2 Fair ☐ 3 Poor ☐ 4 Very poor ☐ 5

**B10 <u>Before your first biopsy,</u> did you have any of the following symptoms? If so, please tell us to what extent, you were affected by each.**

**a. Urinary symptoms**

i) Weak urinary stream

Not at all ☐ 1 Yes, mild ☐ 2 Yes, moderate ☐ 3 Yes, severe ☐ 4

ii) Urgency or incontinence *(leakage of urine)*

Not at all ☐ 1 Yes, mild ☐ 2 Yes, moderate ☐ 3 Yes, severe ☐ 4

iii) Feeling of incomplete bladder emptying

Not at all ☐ 1 Yes, mild ☐ 2 Yes, moderate ☐ 3 Yes, severe ☐ 4

iv) Getting up at night to go to the toilet

Not at all ☐ 1 Yes, mild ☐ 2 Yes, moderate ☐ 3 Yes, severe ☐ 4

**b. Pain while urinating** Not at all ☐ 1 Yes, mild ☐ 2 Yes, moderate ☐ 3 Yes, severe ☐ 4

**c. Impotence or erectile dysfunction** ***(unable to get or sustain an erection)***

Not at all ☐ 1 Yes, mild ☐ 2 Yes, moderate ☐ 3 Yes, severe ☐ 4

**d. Loss of interest in sex** Not at all ☐ 1 Yes, mild ☐ 2 Yes, moderate ☐ 3 Yes, severe ☐ 4

**e. Back pain** Not at all ☐ 1 Yes, mild ☐ 2 Yes, moderate ☐ 3 Yes, severe ☐ 4

**f. Blood in urine** Yes ☐ 1 No ☐ 2

**g. Other** Yes ☐ 1 No ☐ 2

ii) ***If Other,*** please describe ☐

**B11** **Before your first biopsy**, did you have any of the following health problems? *(Tick yes OR no for each)*

| | Yes | No | | Yes | No |
|---|---|---|---|---|---|
| a) Heart disease | ☐ 1 | ☐ 2 | f) High blood pressure | ☐ 1 | ☐ 2 |
| b) Lung disease | ☐ 1 | ☐ 2 | g) Diverticular disease | ☐ 1 | ☐ 2 |
| c) Stroke | ☐ 1 | ☐ 2 | h) Other bowel problems (eg. constipation/diarrohea) | ☐ 1 | ☐ 2 |
| d) Diabetes | ☐ 1 | ☐ 2 | i) Cancer | ☐ 1 | ☐ 2 |
| e) Depression | ☐ 1 | ☐ 2 | j) Other | ☐ 1 | ☐ 2 |

***If Other,*** **please describe**

☐

**B12** **Before your first biopsy,** were you given information to help you feel prepared? *(e.g. what the biopsy would involve, what you should or shouldn't do before your biopsy?) (Please tick ONE box)*

- Yes, I was given enough information ☐ 1
- Yes, but I would have liked more information ☐ 2
- No, I was not given any information, but I did not want/need any ☐ 3
- No, I was not given any information, but I would have liked some ☐ 4

**B13** **In the past,** have you ever failed to attend for a prostate biopsy appointment that was organised for you?

Yes ☐ 1 No ☐ 2

**B14** **In the past,** have you ever declined to have a biopsy following a raised PSA test? Yes ☐ 1 No ☐ 2

# Section C Your Recent Biopsy

***In this section we would like to find out how the prostate biopsy was for you. Please answer each question by putting a ✓ in the appropriate box or using the space provided.***

**C1 a. Including your most recent biopsy, how many prostate biopsies have you had?**

One, this was my first ☐ 1 Two ☐ 2 Three or more ☐ 3

**b. When did you have your first prostate biopsy?**

d: ☐☐ m: ☐☐ y: ☐☐☐☐ Can't remember ☐

**c. In which hospital did you have your first prostate biopsy?**

☐

**C2 If you have had more than one biopsy, please tell us …**

**a. When did you have your most recent prostate biopsy?**

d: ☐☐ m: ☐☐ y: ☐☐☐☐ Can't remember ☐

**b. In which hospital did you have your most recent prostate biopsy?**

☐

**C3 We want to find out how long you waited for your biopsy/biopsies.** ***(Please answer the ONE question that applies to you)***

**If you have had one biopsy:** How long was it between being told that your PSA was raised and attending the hospital for your biopsy? ☐☐ weeks ***Or*** ☐☐ months

**OR**

**If you have had more than one biopsy:** How long was it between being told that you needed another biopsy and attending for it? ☐☐ weeks ***Or*** ☐☐ months

***The following questions are about your most recent biopsy.***

**C4 For your most recent biopsy appointment, did you bring someone with you to the hospital?**

Yes ☐ 1 No ☐ 2

**C5 Did the doctor or nurse explain to you that the biopsy might be painful?** ***(Please tick ONE box)***

Yes, I was prepared for the level of pain ☐ 1

Yes, but I was unprepared for the level of pain ☐ 2

Yes, but the pain was less than I expected ☐ 3

No explanation was given ☐ 4

**C6 Were you offered a local anaesthetic?** Yes ☐ 1 No ☐ 2 Can't remember/Don't know ☐ 3

**C7 Did you have a clean prep to empty your bowel?** Yes ☐ 1 No ☐ 2 Can't remember/Don't know ☐ 3

**C8 How many tissue samples (cores) were taken during your biopsy?** 6 ☐ 1 9 ☐ 2 12 ☐ 3 Don't know ☐ 4

**C9 How long did you spend at your biopsy appointment?** ***(Please include the time that you spent waiting AND the time you spent with the doctor and/or nurse.)*** ☐☐ hours **Or** ☐☐ minutes

**C10 Below are some statements men use to describe having a prostate biopsy. Please read each statement and tell us the extent you agree or disagree with it.** ***(Tick one box for EACH statement.)***

| | ***Strongly agree*** | ***Agree*** | ***Disagree*** | ***Strongly disagree*** |
|---|---|---|---|---|
| a) It made me feel **reassured** | ☐ 1 | ☐ 2 | ☐ 3 | ☐ 4 |
| b) It was **embarrassing** | ☐ 1 | ☐ 2 | ☐ 3 | ☐ 4 |
| c) It was **necessary** | ☐ 1 | ☐ 2 | ☐ 3 | ☐ 4 |
| d) It was **painful** | ☐ 1 | ☐ 2 | ☐ 3 | ☐ 4 |
| e) It was **not a big deal** | ☐ 1 | ☐ 2 | ☐ 3 | ☐ 4 |
| f) It made me feel **anxious** | ☐ 1 | ☐ 2 | ☐ 3 | ☐ 4 |
| g) It was **undignified** | ☐ 1 | ☐ 2 | ☐ 3 | ☐ 4 |
| h) It is **something I regret** | ☐ 1 | ☐ 2 | ☐ 3 | ☐ 4 |
| i) It was **stressful** | ☐ 1 | ☐ 2 | ☐ 3 | ☐ 4 |
| j) It made me feel **scared** | ☐ 1 | ☐ 2 | ☐ 3 | ☐ 4 |
| k) It gave me **peace of mind** | ☐ 1 | ☐ 2 | ☐ 3 | ☐ 4 |
| l) It was **unpleasant** | ☐ 1 | ☐ 2 | ☐ 3 | ☐ 4 |
| m) It **took less time** than I thought it would | ☐ 1 | ☐ 2 | ☐ 3 | ☐ 4 |
| n) It was **exhausting** | ☐ 1 | ☐ 2 | ☐ 3 | ☐ 4 |
| o) It was **not as bad** as I thought it would be | ☐ 1 | ☐ 2 | ☐ 3 | ☐ 4 |
| p) It was **uncomfortable** | ☐ 1 | ☐ 2 | ☐ 3 | ☐ 4 |
| q) It made me feel **worried** | ☐ 1 | ☐ 2 | ☐ 3 | ☐ 4 |
| r) It is something **I am glad I had done** | ☐ 1 | ☐ 2 | ☐ 3 | ☐ 4 |

**C11 a. How many miles did you travel to hospital where you had your biopsy?** ☐ miles

**b. How long did it take you to get to hospital?** ☐ minutes

**c. How did you travel to the hospital?** ***(Please choose ONE option to describe how you travelled for the main part (longest in terms of distance) of your journey)***

| | | | |
|---|---|---|---|
| Walking | ☐ 1 | Taxi | ☐ 5 |
| Car | ☐ 2 | Hospital mini bus | ☐ 6 |
| Bus | ☐ 3 | Tram | ☐ 7 |
| Train | ☐ 4 | Other *(eg plane, bike)* | ☐ 8 |

***If Other,*** **please describe**

☐

**d. If you used public transport or taxis, how much was the fare?** € ☐

**e. If you travelled by car, how much did you pay for fuel (one way) and parking?** ***(Please be as exact as you can)***

Petrol/Diesel € ☐ Parking € ☐

# Section D After Your Biopsy

***In this section, we would like to find out how you have been since your biopsy. Please answer each question by putting a ✓ in the appropriate box or using the space provided.***

**D1 a. Have you been told the result of your most recent biopsy?** Yes ☐1 No ☐2 **IF NO, GO TO D2**

***If Yes,***

**b. When did you get the result?** d: ☐☐ m: ☐☐ y: ☐☐☐☐

**c. What was your result? *(Please tick ONE option)***

Positive for prostate cancer ☐1

Negative, clear of prostate cancer ☐2

Uncertain, need further tests ☐3

Don't know ☐4

**d. If your biopsy was positive for cancer, have you been told the extent or stage of the cancer or how far it has spread? *(eg. the Gleason grade eg. 3+3)***

Yes ☐1 No ☐2 Don't know ☐3

**If Yes, please describe**

☐

**D2 Were you aware that you might have some after-effects following your biopsy?** Yes ☐1 No ☐2

**D3 a. After your most recent biopsy, did you have a raised temperature or fever?**

Yes ☐1 No ☐2 **IF NO, GO TO D4**

**b. How long did the pain last?** ☐ hours ☐ days ☐ weeks

***If Yes,***

**c. At its worst, was your raised temperature or fever?**

Very mild ☐1 Mild ☐2 Moderate ☐3 Severe ☐4 Very severe ☐5

**d. Did you consult any of the following about your raised temperature or fever? *(Please tick yes OR no for each)***

| | Yes | No | | Yes | No |
|---|---|---|---|---|---|
| i) GP | ☐1 | ☐2 | iv) Chemist/pharmacist | ☐1 | ☐2 |
| ii) Hospital doctor | ☐1 | ☐2 | v) Hospital Accident & Emergency Department *(A&E)* | ☐1 | ☐2 |
| iii) Hospital nurse/nurse specialist | ☐1 | ☐2 | | | |

**e. Did you take any medication for your raised temperature or fever?** Yes ☐1 No ☐2

**IF NO, GO TO D4**

**f. *If Yes,* what medication did you take?**

☐

**g. How much did you pay for this medication?** € ☐

**D4** a. **<u>After your most recent biopsy,</u> did you have pain?** Yes ☐1 No ☐2 **IF NO, GO TO D5**

b. ***If Yes*, where or when did you experience pain? *(Please tick yes OR no for each)***

| | Yes | No | | Yes | No |
|---|---|---|---|---|---|
| i) I had pain at the biopsy site | ☐1 | ☐2 | I had pain while ejaculating | ☐1 | ☐2 |
| ii) I had pain while urinating | ☐1 | ☐2 | I had pain elsewhere | ☐1 | ☐2 |

**If pain elsewhere describe**

c. **How long did the pain last?** ☐ hours ☐ days ☐ weeks

d. **At its worst, was your pain?**

Very mild ☐1 Mild ☐2 Moderate ☐3 Severe ☐4 Very severe ☐5

e. **Did you consult any of the following about your pain? *(Please tick ALL that apply)***

| | Yes | No | | Yes | No |
|---|---|---|---|---|---|
| i) GP | ☐1 | ☐2 | iv) Chemist/pharmacist | ☐1 | ☐2 |
| ii) Hospital doctor | ☐1 | ☐2 | v) Hospital Accident & Emergency Department (A&E) | ☐1 | ☐2 |
| iii) Hospital nurse/nurse specialist | ☐1 | ☐2 | | | |

f. **Did you take any medication for the pain?** Yes ☐1 No ☐2 **IF NO, GO TO D5**

g. ***If Yes*, what medication did you take?**

h. **How much did you pay for this medication?** €

**D5** a. **<u>After your most recent biopsy,</u> did you have any bleeding?** Yes ☐1 No ☐2 **IF NO, GO TO D6**

b. ***If Yes*, where was the blood? *(Please circle ALL that apply)***

| | Yes | No |
|---|---|---|
| i) in Urine | ☐1 | ☐2 |
| ii) in semen/ejaculate | ☐1 | ☐2 |
| iii) from rectum/in stools | ☐1 | ☐2 |

c. **How long did the bleeding last?** ☐ hours ☐ days ☐ weeks

d. **At its worst, was your bleeding?**

Very mild ☐1 Mild ☐2 Moderate ☐3 Severe ☐4 Very severe ☐5

e. **Did you consult any of the following about your bleeding? *(Please tick yes OR no for each)***

| | Yes | No | | Yes | No |
|---|---|---|---|---|---|
| i) GP | ☐1 | ☐2 | iv) Chemist/pharmacist | ☐1 | ☐2 |
| ii) Hospital doctor | ☐1 | ☐2 | v) Hospital Accident & Emergency Department (A&E) | ☐1 | ☐2 |
| iii) Hospital nurse/nurse specialist | ☐1 | ☐2 | | | |

f. **Did you take any medication for the bleeding?** Yes ☐1 No ☐2 **IF NO, GO TO D6**

g. ***If Yes*, what medication did you take?**

h. **How much did you pay for this medication?** €

**D6** a. __After your most recent biopsy,__ did you experience erectile dysfunction *(problems getting and/or maintaining an erection)?*

Yes ☐ 1 No ☐ 2 **IF NO, GO TO D7**

b. ***If Yes,*** **how long did the erectile dysfunction last?** ☐ once only ☐ days ☐ weeks

**c. At its worst, was your erectile dysfunction?**

Very mild ☐ 1 Mild ☐ 2 Moderate ☐ 3 Severe ☐ 4 Very severe ☐ 5

**d. Did you consult any of the following about erectile dysfunction?** ***(Please tick yes OR no for each)***

| | Yes | No | | | Yes | No |
|---|---|---|---|---|---|---|
| i) GP | ☐ 1 | ☐ 2 | iv) | Chemist/pharmacist | ☐ 1 | ☐ 2 |
| ii) Hospital doctor | ☐ 1 | ☐ 2 | v) | Hospital Accident & Emergency Department (A&E) | ☐ 1 | ☐ 2 |
| iii) Hospital nurse/nurse specialist | ☐ 1 | ☐ 2 | | | | |

**e. Did you take any medication for the erectile dysfunction?** Yes ☐ 1 No ☐ 2 **IF NO, GO TO D7**

**f.** ***If Yes*****, what medication did you take?**

☐

**g. How much did you pay for this medication?** €

**h. Have you been sexually active** ***(with or without intercourse)*** **since your most recent biopsy?**

Yes ☐ 1 No ☐ 2

**D7** a. __After your most recent biopsy,__ urinary retention *(problems emptying your bladder completely)?*

Yes ☐ 1 No ☐ 2 **IF NO, GO TO D8**

**b.** ***If Yes,*** **how long did the urinary retention last?** ☐ hours ☐ days ☐ weeks

**c. At its worst, was your urinary retention?**

Very mild ☐ 1 Mild ☐ 2 Moderate ☐ 3 Severe ☐ 4 Very severe ☐ 5

**d. Did you consult any of the following about your urinary retention?** ***(Please tick yes OR no for each)***

| | Yes | No | | | Yes | No |
|---|---|---|---|---|---|---|
| i) GP | ☐ 1 | ☐ 2 | iv) | Chemist/pharmacist | ☐ 1 | ☐ 2 |
| ii) Hospital doctor | ☐ 1 | ☐ 2 | v) | Hospital Accident & Emergency Department (A&E) | ☐ 1 | ☐ 2 |
| iii) Hospital nurse/nurse specialist | ☐ 1 | ☐ 2 | | | | |

**e. Did you take any medication for your urinary retention?** Yes ☐ 1 No ☐ 2 **IF NO, GO TO D8**

**f.** ***If Yes,*** **what medication did you take?**

☐

**g. How much did you pay for this medication?** €

**D8 a. Were you admitted to hospital because of complications following your most recent biopsy?**

Yes ☐ 1 No ☐ 2

***If Yes,***

**b. how many nights did you stay in hospital?** ☐

**c. did you have to pay for your stay in hospital?** Yes ☐ 1 No ☐ 2

**d. how much did you pay?** € ☐

**D9 a. Did you have any other after-effects following your most recent biopsy?** Yes ☐ 1 No ☐ 2

**b. *If Yes,* please describe**

☐

**D10 Overall, were your after-effects? *(Please tick ONE box)***

- Same as you expected ☐ 1
- Worse than you expected ☐ 2
- Not as bad as you expected ☐ 3
- I did not have any after effects ☐ 4

**D11 To what extent have the after-effects of your biopsy impacted on your sex life? *(Please tick ONE box)***

- Not at all ☐ 1
- A little bit ☐ 2
- Quite a bit ☐ 3
- I did not have any after-effects ☐ 4
- I am not sexually active ☐ 5

**D12 Thinking of the year or so before you had your most recent biopsy, please tell us how important sex is in your life? *(Please tick ONE box)***

- Not at all important ☐ 1
- Slightly important ☐ 2
- Moderately important ☐ 3
- Very important ☐ 4

**D13 If there is anything else you would like to tell us about the impact of your biopsy, or any after-effects, on your life please use the box below.**

☐

# Section E Work

***This section asks about your work. Please answer each question by putting a ✓ in the appropriate box or using the space provided.***

**E1 <u>At the time of your most recent biopsy,</u> which of the following best described you?** ***(Please tick ONE box)***

| | | | |
|---|---|---|---|
| Paid employment | ☐ 1 | Unemployed | ☐ 5 |
| Self employed | ☐ 2 | Unable to work due to permanent sickness/disability | ☐ 6 |
| Retired from employment | ☐ 3 | Voluntary work | ☐ 7 |
| Looking after family/home | ☐ 4 | Other | ☐ 8 |

***If Other,* please describe**

☐

**E2 What do/did you do in your current or most recent job?** ***(Please describe your occupation fully e.g. RETAIL STORE MANAGER, SECONDARY TEACHER, LABOURER, BUILDER, FARMER.)***

**Your occupation**

☐

**E3 What is/was the business or main activity of your employer?** ***(e.g. MAKING COMPUTERS, FOOD WHOLESALE, FARMING. If you were self-employed, please answer in respect of your own business.)***

**Employer's business**

☐

**E4 How many hours do/did you usually work?** ☐ **hours/week**

**E5 If you had not gone to your most recent biopsy appointment, what would you normally have been doing?** ***(Please tick ONE box that best describes your MAIN activity)***

| | | | |
|---|---|---|---|
| Paid work | ☐ 1 | Leisure activities | ☐ 3 |
| Unpaid work | ☐ 2 | Other | ☐ 4 |

***If Other,* please describe**

☐

**E6 If you would have normally been in <u>paid work,</u> did you…** ***(Please tick yes OR no for each)***

| | Yes | No |
|---|---|---|
| Lose any earnings | ☐ 1 | ☐ 2 |
| Have to take annual leave | ☐ 1 | ☐ 2 |
| Have to take flexi time hours | ☐ 1 | ☐ 2 |
| Other | ☐ 1 | ☐ 2 |

***If Other,* please describe**

☐

**E7 When you attended your most recent biopsy appointment did you lose any earnings?**

Yes ☐ 1 No ☐ 2

# Section F How are things NOW

***Please answer each of the following questions circling the number that best applies to you, by putting a ✓ in the appropriate box, or using the space provided.***

**F1 We would like to know whether you have been thinking about your prostate biopsy. Below is a list of statements. Please tell us how often each statement was true for you <u>during the last week.</u>**
***(Please circle ONE number for each statement)***

| During the past week | Not at all | Rarely | Sometimes | Often |
|---|---|---|---|---|
| **a.** I thought about it when I didn't mean to | 1 | 2 | 3 | 4 |
| **b.** I avoided letting myself get upset when I thought about or was reminded of it | 1 | 2 | 3 | 4 |
| **c.** I tried to remove it from my memory | 1 | 2 | 3 | 4 |
| **d.** I had trouble falling asleep or staying asleep because of thoughts about it that came into my mind | 1 | 2 | 3 | 4 |
| **e.** I had waves of strong feelings about it | 1 | 2 | 3 | 4 |
| **f.** I had dreams about it | 1 | 2 | 3 | 4 |
| **g.** I stayed away from reminders of it | 1 | 2 | 3 | 4 |
| **h.** I felt as if it hadn't happened or wasn't real | 1 | 2 | 3 | 4 |
| **i.** I tried not to talk about it | 1 | 2 | 3 | 4 |
| **j.** Pictures about it popped into my head. | 1 | 2 | 3 | 4 |
| **k.** Other things kept making me think about it | 1 | 2 | 3 | 4 |
| **l.** I tried not to think about it | 1 | 2 | 3 | 4 |
| **m.** I was aware that I still had a lot of feelings about it, but I didn't deal with them | 1 | 2 | 3 | 4 |
| **n.** Any reminder brought back feelings about it. | 1 | 2 | 3 | 4 |
| **o.** My feelings about it were kind of numb | 1 | 2 | 3 | 4 |

**F2 These questions are about how you cope with stress. Please tell us if, or how often, you have been doing any of the following <u>in relation to your prostate biopsy</u>. Make sure the answer is as true for you as possible. *(Please circle ONE number on each row.)***

| | Haven't been doing this at all | Doing this a little | Doing this a medium amount | Doing this a lot |
|---|---|---|---|---|
| **a.** I've been turning to work or other activities to take my mind off things | 1 | 2 | 3 | 4 |
| **b.** I've been concentrating my efforts on doing something about the situation I'm in | 1 | 2 | 3 | 4 |
| **c.** I've been saying to myself "this isn't real" | 1 | 2 | 3 | 4 |
| **d.** I've been using alcohol or other drugs to make myself better | 1 | 2 | 3 | 4 |
| **e.** I've been getting emotional support from others | 1 | 2 | 3 | 4 |
| **f.** I've been giving up trying to deal with it | 1 | 2 | 3 | 4 |
| **g.** I've been trying to take action to try to make the situation better | 1 | 2 | 3 | 4 |
| **h.** I've been refusing to believe that it has happened | 1 | 2 | 3 | 4 |
| **i.** I've been saying things to let my unpleasant feelings escape | 1 | 2 | 3 | 4 |
| **j.** I've been getting help and advice from other people | 1 | 2 | 3 | 4 |
| **k.** I've been using alcohol or other drugs to help me get through it | 1 | 2 | 3 | 4 |
| **l.** I've been trying to see it in a different light, to make it seem more positive | 1 | 2 | 3 | 4 |
| **m.** I've been criticizing myself | 1 | 2 | 3 | 4 |
| **n.** I've been trying to come up with a strategy/plan about what to do | 1 | 2 | 3 | 4 |
| **o.** I've been getting comfort and understanding from someone | 1 | 2 | 3 | 4 |
| **p.** I've been giving up the attempt to cope | 1 | 2 | 3 | 4 |
| **q.** I've been looking for something good in what is happening | 1 | 2 | 3 | 4 |
| **r.** I've been making jokes about it | 1 | 2 | 3 | 4 |
| **s.** I've been doing something to think about it less, such as going to the movies, watching TV, reading, daydreaming, sleeping, shopping | 1 | 2 | 3 | 4 |
| **t.** I've been accepting the reality of the fact that it has happened | 1 | 2 | 3 | 4 |
| **u.** I've been expressing my negative feelings | 1 | 2 | 3 | 4 |
| **v.** I've been trying to find comfort in my religion or spiritual beliefs | 1 | 2 | 3 | 4 |
| **w.** I've been trying to get advice or help from other people about what to do | 1 | 2 | 3 | 4 |
| **x.** I've been learning to live with it | 1 | 2 | 3 | 4 |
| **y.** I've been thinking hard about what steps to take | 1 | 2 | 3 | 4 |
| **z.** I've been blaming myself for things that happened | 1 | 2 | 3 | 4 |
| **aa.** I've been praying or meditating | 1 | 2 | 3 | 4 |
| **ab.** I've been making fun of the situation | 1 | 2 | 3 | 4 |

## F3 Mobility

- I have no problems in walking about ☐ 1
- I have slight problems in walking about ☐ 2
- I have moderate problems in walking about ☐ 3
- I have severe problems in walking about ☐ 4
- I am unable to walk about ☐ 5

## F4 Self care

- I have no problems washing or dressing myself ☐ 1
- I have slight problems washing or dressing myself ☐ 2
- I have moderate problems washing or dressing myself ☐ 3
- I have severe problems washing or dressing myself ☐ 4
- I am unable to wash or dress myself ☐ 5

## F5 Usual Activities *(eg. work, study, housework, family or leisure activities)*

- I have no problems doing my usual activities ☐ 1
- I have slight problems doing my usual activities ☐ 2
- I have moderate problems doing my usual activities ☐ 3
- I have severe problems doing my usual activities ☐ 4
- I am unable to do my usual activities ☐ 5

## F6 Pain/Discomfort

- I have no pain or discomfort ☐ 1
- I have slight pain or discomfort ☐ 2
- I have moderate pain or discomfort ☐ 3
- I have severe pain or discomfort ☐ 4
- I have extreme pain or discomfort ☐ 5

## F7 Anxiety/Depression

- I am not anxious or depressed ☐ 1
- I am slightly anxious or depressed ☐ 2
- I am moderately anxious or depressed ☐ 3
- I am severely anxious or depressed ☐ 4
- I am extremely anxious or depressed ☐ 5

**F8 We would like to know how good or bad your health is TODAY**

- This scale is numbered from 0 to 100.
- 100 means the best health you can imagine.
- 0 means the worst health you can imagine.
- Mark an **X** on the scale to indicate how your health is **TODAY.**
- Now, please write the number you marked on the scale in the box below.

**YOUR HEALTH TODAY =** [ ]

**The best health you can imagine**

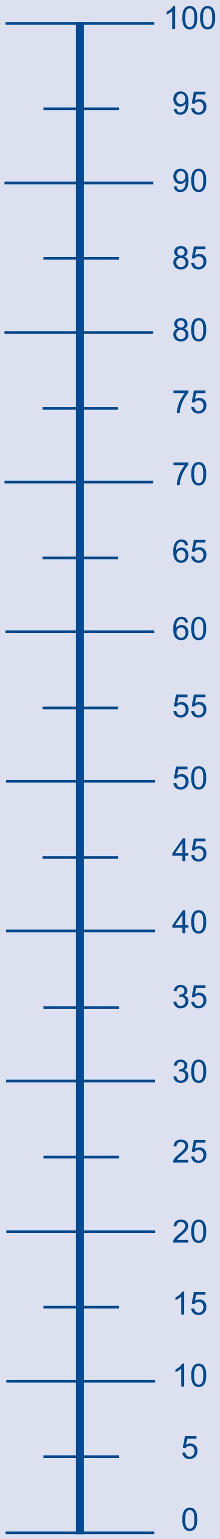


**The worst health you can imagine**

**F9 Thinking about your recent experiences, tell us which of the following statements best describes your views.**

**a. When discussing <u>PSA blood tests</u> with a friend or family member, I would...** ***(Please tick ONE box)***

Strongly recommend it to them ☐ 1

Recommend it to them ☐ 2

Discourage them from having it ☐ 3

Strongly discourage them from having it ☐ 4

**b. When discussing <u>prostate biopsies</u> with a friend or family member, I would...** ***(Please tick ONE box)***

Strongly recommend it to them ☐ 1

Recommend it to them ☐ 2

Discourage them from having it ☐ 3

Strongly discourage them from having it ☐ 4

**F10 Do you think that the availability of PSA testing should be more widely publicised?**

Yes ☐ 1 No ☐ 2 Not sure/No opinion ☐ 3

**F11 All things considered, how satisfied are you with your life as a whole these days?** ***(Please circle ONE number)***

| 1 | 2 | 3 | 4 | 5 | 6 | 7 | 8 | 9 | 10 |
|---|---|---|---|---|---|---|---|---|---|
| *Dissatisfied* | | | | | | | | | *Satisfied* |

**F12 If there is anything else you would like to tell us about your prostate biopsy, or its impact on your life, please use the box below.**

**F13. Would you be willing to be contacted again about future studies relating to prostate investigations?**

Yes ☐ 1 No ☐ 2

# Thank you for completing this important survey.

**Please return it, with your signed consent form, in the pre-paid envelope provided:**

**Dr Frances Drummond, Project coordinator**
**National Cancer Registry, Building 6800, Airport Business Park, Cork.**

**Telephone: 021 4548837; Email: f.drummond@ncri.ie**

Thank you for completing this important survey.

Please return it, with your signed consent form, in the pre-paid envelope provided:
Dr Frances Drummond, Project coordinator
National Cancer Registry, Building 6800, Airport Business Park, Cork.

Telephone: 021 4548837; Email: f.drummond@ncri.ie

PiCTure
Prostate
Investigations: *your experience*
